# Transnasal Marsupialization Using Endoscopic Sinus Surgery for Treatment of Keratocystic Odontogenic Tumor in Maxillary Sinus

**DOI:** 10.1155/2012/281402

**Published:** 2012-09-30

**Authors:** Masafumi Ohki

**Affiliations:** Department of Otolaryngology, Saitama Medical Center, Kawagoe-shi, Saitama 350-8550, Japan

## Abstract

*Objective.* We report the first utilisation of transnasal marsupialization to treat a keratocystic odontogenic tumor in the maxillary sinus of a 37-year-old man. *Case Report.* A 37-year-old man presented with a nasal discharge and right odontalgia. Computed tomography revealed an expanding cystic lesion with a calcificated wall containing an impacted tooth in the right maxillary sinus. The diagnosis was keratocystic odontogenic tumor. Transnasal marsupialization was performed using endoscopic sinus surgery to enlarge the maxillary ostium and remove a portion of the cystic wall. Pathological findings included lining squamous epithelium and inflammation. The remaining tumor shrank, becoming free of infection after surgery, without proliferation. *Conclusion.* Transnasal marsupialization using endoscopic sinus surgery is effective in treating keratocystic odontogenic tumors. It offers minimal surgical invasion and reductive change, making it advantageous for complete removal with fewer complications in the bones and surrounding tissue in the case of secondary surgery.

## 1. Introduction

Odontogenic keratocysts were first reported by Philipsen in 1956 [1]. Odontogenic keratocysts are dentigerous cysts derived from the proliferation of residues of the dental lamina. Odontogenic keratocysts potentially behave aggressively, and the rate of recurrence is high, varying from 0 to 62% according to the various treatments [2]. In 2005, odontogenic keratocysts were reclassified by the World Health Organization as keratocystic odontogenic tumors and defined as benign unicystic or multicystic intraosseous tumors of odontogenic origin [3]. Many different treatments, ranging from simple curettage to highly invasive en block resection with extension margins, have been used. However, there is no consensus on the most effective method. We attempted a minimally invasive marsupialization procedure via a transnasal approach using endoscopic sinus surgery (ESS) for odontogenic keratocysts of the maxilla as a first stage operation. In this paper, we present the first case of a keratocystic odontogenic tumor in the maxillary sinus treated using ESS and discuss the effectiveness of surgery.

## 2. Case Report

**Figure 1:**
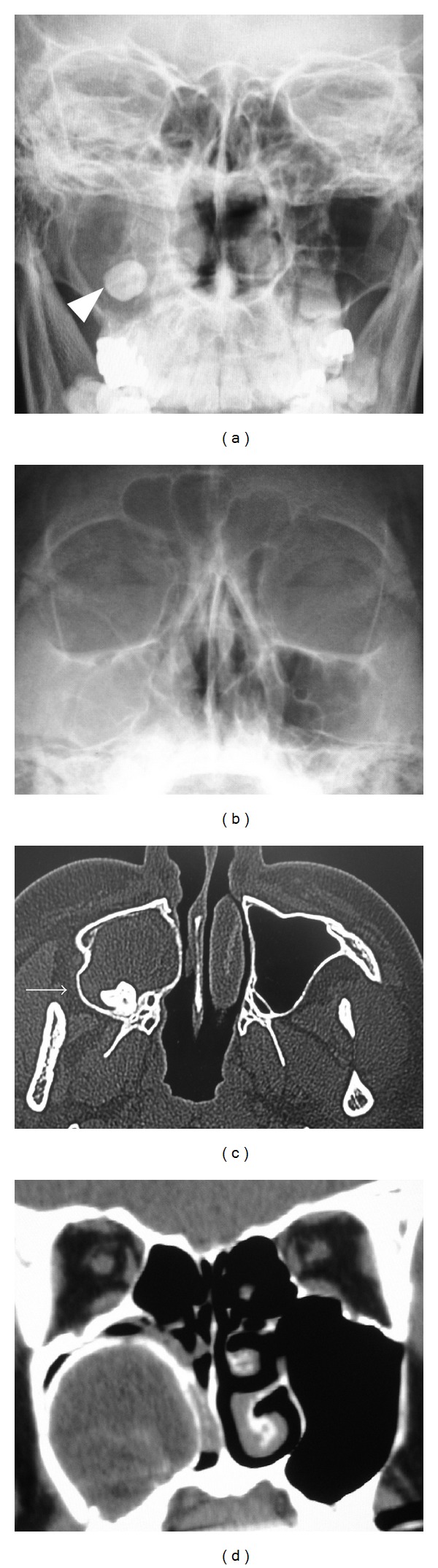
(a) X-ray shows cloudy appearance in the right maxillary sinus including an impacted tooth (arrowhead) in the Caldwell view. (b) X-ray shows cloudy appearance in the right maxillary sinus in the Waters view. (c) Axial view of the CT scan shows a cystic lesion expanding the lateral wall (arrow) of the maxillary sinus containing the impacted tooth inside. (d) Coronal view of the CT scan shows the calcificated cystic wall.

**Figure 2:**
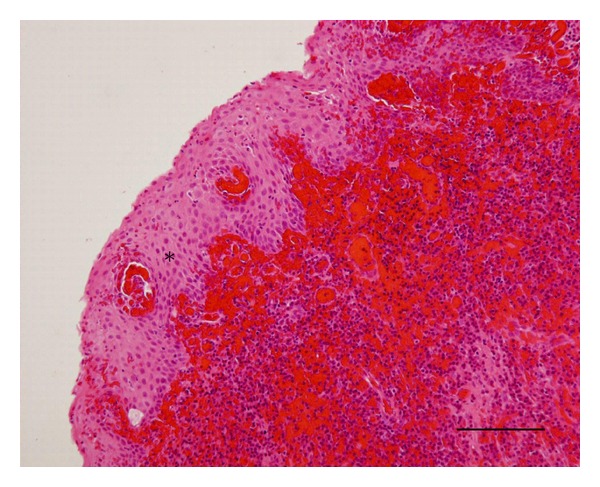
Pathological findings reveal lining squamous epithelium (asterisk) and inflamed tissue with lymphocytes and plasma cells. The bar indicates 100 *μ*m. Hematoxylin and eosin stain.

**Figure 3:**
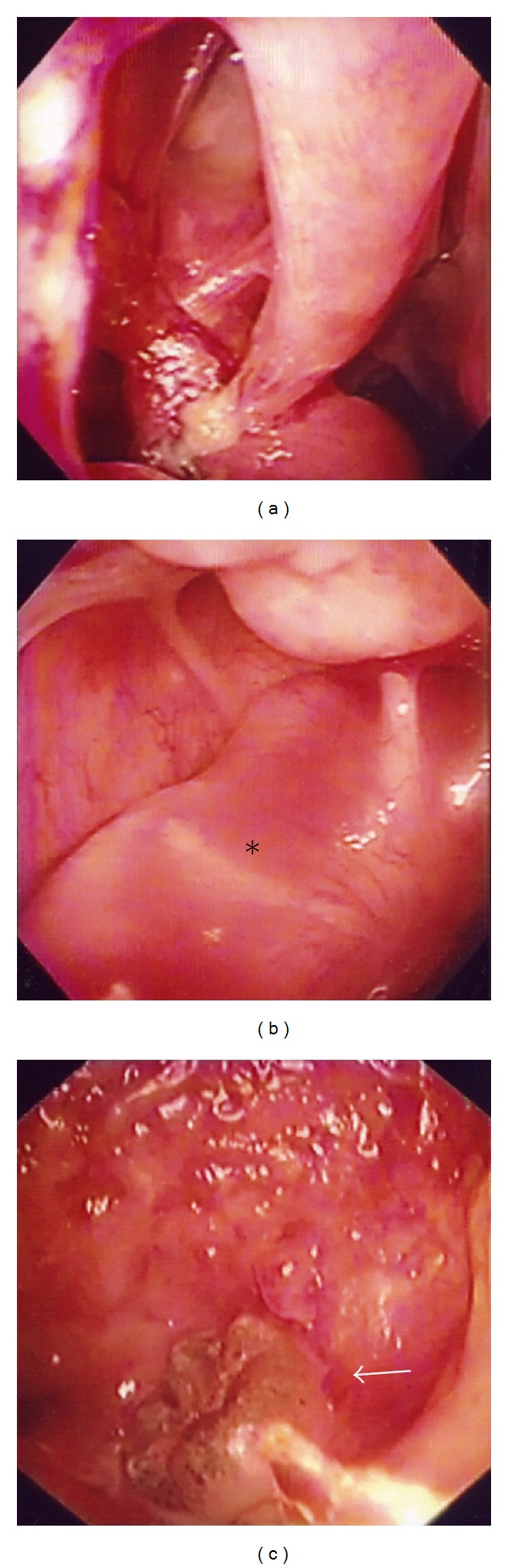
(a) Postoperatively, maxillary sinus is visualized through the middle meatus using a flexible fiberscope. (b) Cystic lesion (asterisk) is shrunken in the maxillary sinus. (c) Arrow shows an impacted tooth inside the cystic lesion.

A 37-year-old man presented with a purulent nasal discharge and odontalgia on the right side of his face. The purulent discharge from the right gingiva began two years earlier and persisted despite dental treatment. The right inferior turbinate was swollen, and the lateral nasal wall protruded. Pus discharged from the right gingiva near the second molar. Dental caries were not detected in his right maxilla. An X-ray revealed a cloudy appearance in the right maxillary sinus including an impacted tooth (Figures 1(a) and 1(b)). The axial and coronal views of the computed tomographic (CT) scans revealed an expanding cystic lesion with an impacted tooth in the right maxillary sinus (Figures 1(c) and 1(d)). The cystic wall was calcificated (Figure 1(d)). The lateral wall of the maxillary sinus was excluded laterally, and the lateral nasal wall was protruded medially. He underwent endoscopic sinus surgery (ESS) on the right side under general anesthesia. The maxillary ostium was enlarged, and the cystic lesion was opened and decompressed, removing caseous material transnasally through endoscopy. The cystic wall was removed as much as possible. Pathological findings revealed lining squamous epithelium and inflamed tissue with lymphocytes and plasma cells comparable with a keratocystic odontogenic tumor (Figure 2). The case was diagnosed as a keratocystic odontogenic tumor on the right maxillary sinus. We recommended complete removal of the keratocystic odontogenic tumor by a transnasal approach or a transoral approach using a gingivobuccal sulcus incision, such as the Caldwell-Luc method. Our proposal was refused, however, because his symptoms disappeared. After the surgery, the maxillary sinus could be visualized through the middle meatus using a flexible fiberscope (Figures 3(a)–3(c)). In addition, the remaining cystic lesion shrank after the surgery (Figure 3(b)), and he was relieved of the purulent nasal discharge and odontalgia completely. The remaining keratocystic odontogenic tumor did not proliferate during the one-year follow-up period.

## 3. Discussion

Keratocystic odontogenic tumors are hypothesized to be low-grade neoplasms supported by abnormal tumor suppressor gene expression [4–6]. Malignant transformation into squamous cell carcinoma is rarely reported, at 0.12% [7]. Enucleation alone leads to the high-recurrence rate of 17 to 56% [8]. To reduce the recurrence rate, various adjunctive therapies, such as curettement of the surrounding tissues, peripheral ostectomy, the use of Carnoy's solution, cryotherapy, and electrocautery, have been attempted [9]. Ghali and Connor advocates that radical treatments for complete removal, that is, en bloc resection with extension margins, lead to 0% recurrence rates [10]. However, radical treatments can potentially damage the inferior orbicular nerve, inferior alveolar nerve, or tooth root [2]. In addition, en bloc resection with extension margins may require reconstruction surgery. Conversely, conservative treatments have also been reported, such as decompression using a drainage tube or transoral marsupialization, which internalizes the cyst through the surgical window between the buccal mucosa and the cystic wall [2, 8, 11]. These conservative surgeries can preserve almost all bone structures, other than those surrounding the surgical window. The cystic lesion usually shrinks after decompression using a drainage tube or transoral marsupialization [2]. In cases of secondary surgery to completely remove keratocystic odontogenic tumors, subsequent resection after decompression is easier to achieve a lower recurrence rate, fewer complications in the bone, and less surrounding tissue destruction [2]. Moreover, in some cases, decompression leads to dedifferentiation of the keratocystic odontogenic tumor epithelium [2, 12]. In addition, inflammation is considered to induce metaplastic changes in epithelial cells [13]. Therefore, controlling the infection and maintaining decompression of the lesion are crucial for reducing the aggressiveness and recurrence of keratocystic odontogenic tumors. Marsupialized tumors are often less prone to infection because the inside of the tumor, which is frequently the focus of the infection, can be drained. In the case of a large tumor, staged operation is advantageous for reduction of complication risks. For the first stage of the operation, marsupialization is undergone. After the tumor is reduced in size, a secondary operation of complete resection should be scheduled. If keratocystic odontogenic tumors exist in the maxilla, which is adjacent to the nasal cavity, the transnasal approach using ESS is considered suitable for marsupialization. 

We used a novel strategy of transnasal marsupialization using the ESS technique for keratocystic odontogenic tumors to create a surgical window through the artificially enlarged maxillary natural ostium, instead of through the buccal mucosa. Transnasal marsupialization does not require drainage tubing or self-treatment of the surgical window, and food impaction does not occur. The microdebrider is a useful tool to remove cystic wall and enlarge the maxillary ostium in transnasal marsupialization using ESS technique [14]. In addition, infection is relieved because the natural sinus clearance mechanism reduces bacterial contamination of the maxilla. In our case, infection was relieved, and the lesion shrank after transnasal marsupialization in the same manner as Marker et al.'s [2] transoral marsupialisation.

Otolaryngologists are familiar with ESS. The lesion could be visualized transnasally using flexible fiberscopy. Another benefit is the probable shrinkage of the cyst in a similar manner as that in transoral marsupialization, as observed in our case. Keratocystic odontogenic tumors may dedifferentiate into normal epithelium after transnasal marsupialization. However, remnants of the keraticystic odontogenic tumor may lead to recurrence. Therefore, once the lesion is sufficiently reduced in size, definitive surgery is recommended. Shrunken and less aggressive lesions are advantageous for complete removal in secondary surgery, especially in cases of large maxillary keratocystic odontogenic tumor. Presently, endoscopic sinus surgery is performed for sinonasal inverted papillomas [15]. To remove inverted papillomas in maxillary sinus, endoscopic medial maxillectomy is effective. The same method of endoscopic medial maxillectomy will enable complete resection of keratocystic odontogenic tumors. Combination therapy of Carnoy's solution or cryotherapy in addition to surgery is considered to better reduce recurrence rates. After endoscopic medial maxillectomy is performed, the maxillary sinus is easily visible using flexible optic scope. Therefore, this approach also benefits follow-up procedures.
